# Acceptability and usability of oral fluid-based HIV self-testing among female sex workers and men who have sex with men in Morocco

**DOI:** 10.1186/s12889-022-14632-5

**Published:** 2022-12-05

**Authors:** Amal Ben Moussa, Ouijdane Belhiba, Fatima Zahra Hajouji, Amina El Kettani, Mohammed Youbi, Kamal Alami, Boutaina El Omari, Lahoucine Ouarsas, Mehdi Karkouri

**Affiliations:** 1 Association de Lutte Contre le Sida (ALCS), Casablanca, Morocco; 2 Community-based Research Laboratory, Coalition PLUS, Pantin, France; 3 Direction de l’Epidémiologie et de Lutte contre les Maladies (DELM), Rabat, Morocco; 4 Joint United Nations Programme on HIV and AIDS (UNAIDS), Rabat, Morocco

**Keywords:** HIV, Self-testing, Vulnerable populations, Acceptability, Feasibility, Usability, Morocco

## Abstract

**Background:**

In 2020, almost 20% of people living with HIV (PLHIV) in Morocco are still unaware of their HIV status. Under these circumstances, HIV self-test (HIVST) can be an efficient additional tool for improving the testing rates in Morocco and reaching the first objective of the UNAIDS 95–95-95 goal. ALCS, a Community-based organization, involved in HIV Testing since 1992, and the Ministry of Health of Morocco conducted, a study on the acceptability and usability of HIVST among Female sex workers (FSW) and MSM (men who have sex with men), using a salivary rapid test. To our knowledge, this is the first study in Morocco exploring these parameters.

**Methods:**

We conducted a pilot study on the usability of the OraQuick HIV-1/2 salivary self-test among MSM and FSW visiting the ALCS centers for standard HIV rapid testing in five Moroccan cities.

Participants chose whether or not to be assisted by lay provider HIV testing. The counselors sampled them to perform a standard rapid test and then invited them to a private room to perform the HIV self-test simultaneously. In addition, a questionnaire was administered to collect socio-demographic data and to assess their opinion about the usability of the salivary HIVST.

**Results:**

Our study was carried out for 5 months and included 492 participants (257 MSM and 233 FSW). The average age of the participants was 29 years among MSM vs 34 years among FSW. The FSW have a lower educational level, 28,8% of them are Illiterate vs. 6,1% of the MSM.

Only 18% of participants were aware of the existence of the HIVST, nevertheless, we recorded a very high rate of acceptability (90,6%) of the HIVST. Performing the HIVST was deemed very easy for 92,2% of MSM versus 80,6% of FSW. Although it was found very difficult for six participants, including five FSW, 4 of them could not read or write. Overall, the study registered a high HIV positivity rate (3,8%) and 100% of concordance between HIVST participants’ interpretation and standard HIV testing performed by ALCS lay provider HIV testing.

**Conclusion:**

Our study shows very high acceptability of HIVST among FSWs and MSM in Morocco, HIV self-testing is still unknown by key populations in Morocco, and the low level of education of FSWs may be a barrier to the use of this test, but with the proposed assistance and adapted demonstration tools, the HIV self-testing will certainly improve access to testing in Morocco.

## Introduction

More than forty years after the discovery of the first case, HIV and Aids remain a global health crisis. In 2020, 37.7 million people were living with Human Immunodeficiency Virus (HIV) worldwide, with 1.5 million new HIV infections, and 680,000 AIDS-related deaths [1].

To reach the UNAIDS 95–95-95 target, meaning 95% of all seropositive people would know of their HIV status, 95% of them would receive antiretroviral therapy and 95% of those would suppress their viral load, in order to end the AIDS epidemic by 2030, HIV testing is the key and the first step of the HIV care cascade [2].

That target is a challenging goal for all countries worldwide [3], and particularly for HIV testing. In the world, there are nearly 6.1 million [4.9 million–7.3 million] people who still did not know their HIV serological status in 2020 [4].

The main barriers to HIV testing are stigma, discrimination, lack of confidentiality, access to testing sites, and poverty, particularly among key high-risk populations, such as female sex workers (FSW) and men who have sex with men (MSM) [5, 6].

HIV self-testing (HIVST) is an innovative tool. It’s an immunoassay test performed manually for the qualitative detection of antibodies to HIV-1 and HIV-2 in human oral fluid. An oral fluid specimen is collected using the flat pad on the test device, followed by the insertion of the test device into the vial of developer solution. A positive test result in the apparition of reddish-purple line indicating the presence of antibodies to HIV-1 and/or HIV-2 in the specimen. The test results are interpreted after 20 minutes but not more than 40 minutes after the introduction of the test device into the developer solution containing the test specimen.

HIVST and effective way, that can greatly increase the uptake of testing for people who are frequently at risk of HIV infection, especially those who have little or no access to testing due to barriers to access [7, 8]. Since 2016, the World Health Organization (WHO) has recommended the use of HIV self-testing as a safe, accurate to reach people who might not otherwise get tested, including people from key populations [9].

Internationally, and in response to WHO guidelines, in 2020, 78 countries have self-testing policies, and 41 have implemented self-testing. More and more countries are adopting HIV self-testing policies and introducing self-testing [10].

On a regional scale, UNITAID has set up two major projects, “ATLAS “and “STAR”, aiming to introduce, promote and expand HIV self-testing in Africa [11, 12]. Several recent studies conducted in sub-Saharan Africa have demonstrated high acceptability and uptake of HIVST such as in Benin [13], Uganda [14, 15], Nigeria [16], Malawi [17], Kenya [18], and Rwanda [19].

However, there is a lack of data on the acceptability of HIV self-testing in the MENA (Middle East and North Africa) region which has the lowest HIV prevalence in the world (less than 0,1%), but the fastest growing epidemic due to the increase in the number of new infections and deaths related to AIDS. Indeed with With 20,000 new infections in 2019, the region had recorded a 25% increase compared to 2010 [20]. To date, HIV self-testing kits are available in Iran since 2018 and Morocco since 2019 [21]. In Mauritania, a pilot study on the distribution of HIV self-testing kits is underway [22]. Libya, Algeria, Somalia, and Sudan are in the process of developing self-diagnosis policies [20].

In Morocco HIVST was first introduced on September 2019. At the period of this study, it was not yet authorized to sale. It was only available in the frame of “Autotest-Maroc” a demonstration project on acceptability and feasibility of HIV self-testing among key populations carried out by Association de Lutte Contre le Sida (ALCS) in partnership with the Ministry of Health, with the support of Global Fund and UNAIDS.

At the end of 2020, Morocco had an estimated 22,000 People living with HIV (PLHIV), and HIV prevalence in the general population is estimated to be 0,08% but it’s 1.7 among FSW, and 4.9 among MSM [23]. The Kingdom has made significant progress in HIV testing, thanks to the expansion of testing centers and the diversification of the offer, however almost 20% of people living with HIV (PLHIV) in Morocco are still unaware of their HIV status [23]. HIV self-testing could be a good opportunity to help fill this gap.

This study aims to explore the acceptability, and usability of HIVST among FSW and MSM, using OraQuick Rapid HIV-1/2 Antibody self-test HIV and according to recent WHO recommendations [24].

## Methods

The usability evaluation of the OraQuick Rapid HIV-1/2 Antibody Test is a multicenter study performed between September 2019 to January 2020 in five Moroccan cities: Agadir, Casablanca, Marrakech, Rabat, and Tangiers.

To assess the acceptability of oral HIVST, we considered the willing to buy and use of HIVST if it’s available. The usability was assessed by the degree of ease of use and interpret of the test. We also examined the key population’s knowledge about HIV self-testing and asking for the price that they can spend to buy it if authorized to sale in future.

### Study population and design

A convenience sample of 500 participants, was planned and distributed according to each city’s active file.

The participants’ recruitment has occurred in ALCS centers. The suggestion to participate was done for each beneficiary meeting the followed eligibility criteria: to self-identify as a FSW or a MSM, aged 18 years or older, requesting HIV testing at the ALCS centers during the study period, and to be able to provide informed consent. PLHIV and Pre-Exposure Prophylaxis (PrEP) users were excluded from this study, since OraQuick Rapid HIV-1/2 Antibody Test is not suitable for people on ARVs because of the risk of false negatives.

**Fig. 1:**
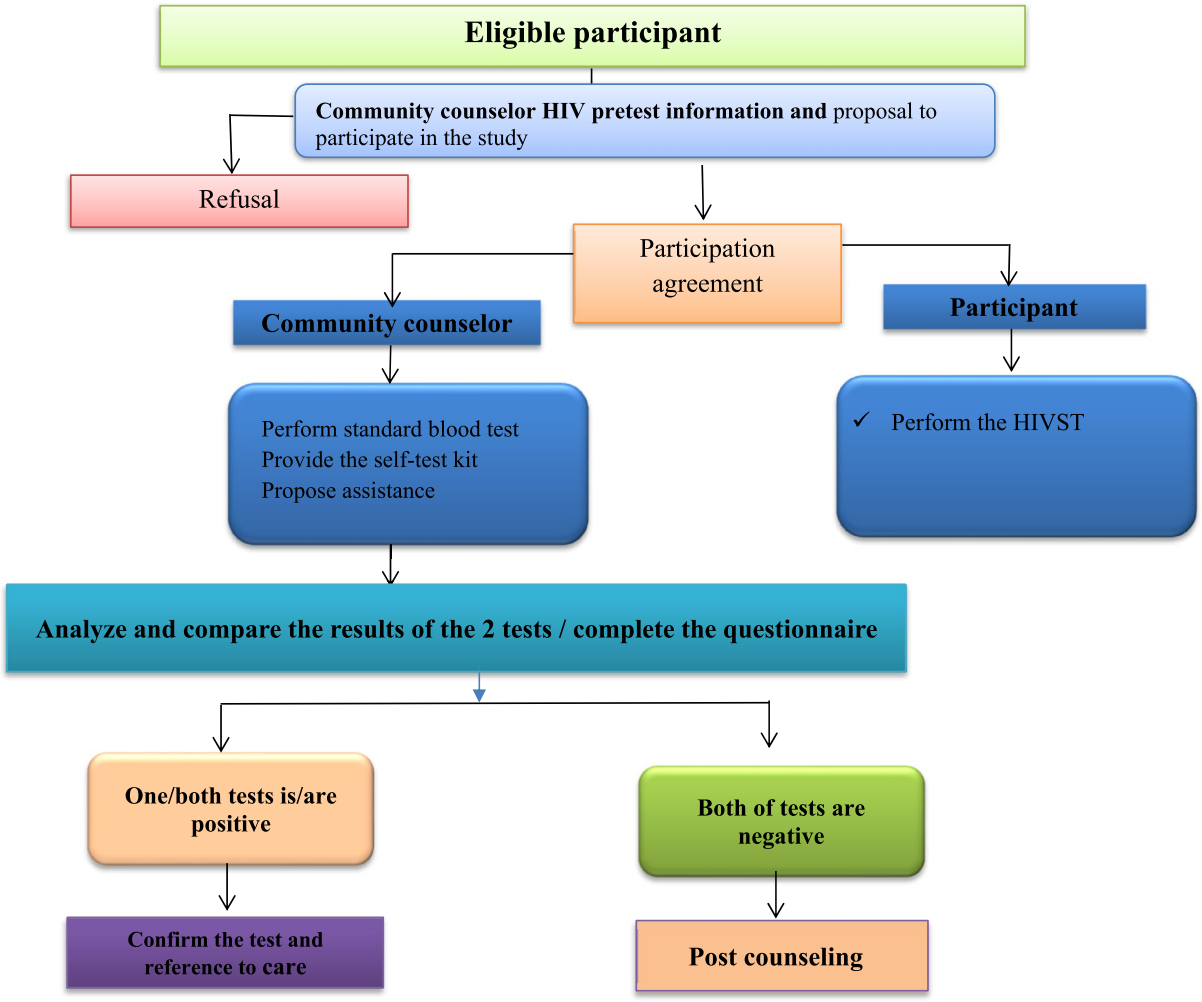
Participant flow

As shown in Fig. 1, all participants were provided with a salivary HIVST, OraQuick Rapid HIV-1/2 Antibody Test that they used on their own in a private room in the ALCS center and self-reported the result. In parallel, rapid standard blood testing was performed by one of the ALCS lay provider HIV testing. Instructions and assistance on using the oral HIV self-testing were also offered to participants. Lay provider HIV testing was requested to check the salivary test and report the result within 20 minutes after the participant performed it.

After completing the self-test, the lay provider HIV testing administrated a questionnaire to the participant and compared the results obtained by the two tests. When a participant has one or both tests positive, he was accompanied to a public care center by a Navigator to confirm the result and benefit from care and support service.

### Participant involvement

The participants and members of the communities MSM and FSW were involved at several stages of this study, including the formative research which allowed us to finalize the design, the questionnaire translation, validation, data collection and results dissemination.

### Data collection tool

A questionnaire was administered face to face by trained interviewers to eligible participants. The interviewers were community counselors who are also HIV testing lay providers in ALCS. The data collected was around socio-demographic features including, sex, age, sexual orientation, relationship status, employment status, education level, and to assess participants’ knowledge of HIV self-testing and their opinion on the usability of the HIV self-testing.

### HIV test used for self-testing

The OraQuick Rapid HIV-1/2 Antibody t self-test HIV (OraSure Technologies, USA) was used to assess the usability of oral HIV self-testing.

This qualitative test detects antibodies to the HIV types 1 and 2 (HIV-1/2) in human oral fluid. It is read visually and provides results in 20 minutes. Moreover, this assay has a concordance of more than 99% with Western blot confirmation.

### Data analysis

The software EpiData was used to collect and manage data. Categorical variables are specified as proportions, with percent and confidence intervals (95%). Quantitative variables are represented by means with IQR.

Factors associated with having any difficulty to perform HIVST were assessed using binary logistic regression. Variables with a *p*-value lower than 0.20 in the univariable analysis were considered eligible to enter the multivariable model. In the multivariable analysis. A backward procedure based on the Likelihood Ratio Chi-2 test was used to select variables for the final model. Statistical analysis was carried out using Stata/SE 14.0 software (StataCorp LP, College Station, USA). Given the small number of transgender people (2 persons), they are not included in this analysis.

## Results

### Participant’s characteristics

**Fig. 2:**
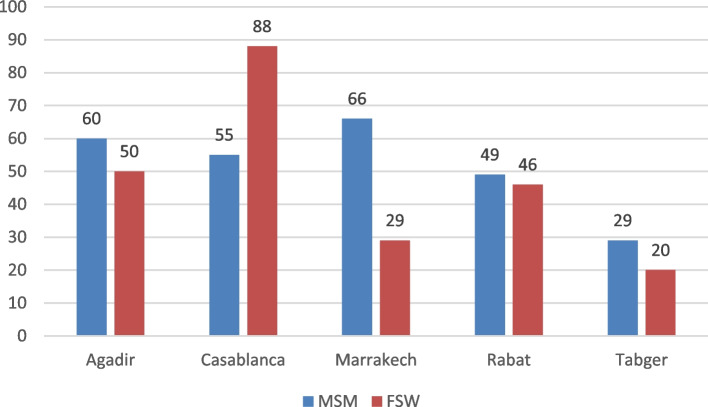
Recruitment of participants in each city

This pilot study was conducted in 5 major cities in Morocco, Agadir, Casablanca, Marrakech, Rabat, and Tangier, lasted 5 months. Eight of the 500 people to whom we suggest to participate to study refused. The included 492 participants, were 259 MSM and 233 FSW. “Fig. 2“.

The median age of participants was 27 years (IQR = 9) for MSM vs. 35 years (IQR = 13) for FSWs. The youngest and oldest participants were MSM at 18 and 76 years, respectively.

Regarding the sexual orientation of the population, we report that 73% of MSM self-identified as homosexual and 26,3% bisexual. Moreover, 90.6% of FSW self-identified as heterosexual.

The educational level and the professional situation in MSM were better compared to in FSW. Indeed, 28,8% of the FSW were illiterate vs. 6,1% of the MSM, and 79% of the FSW were unemployed or occasionally employed vs. 44% of the MSM.

We report that MSM were predominantly single and nearly half of FSW were divorced or separated. All data are summarized in Table 1.

**Table 1:** Sociodemographic characteristics of study participants (MSM and FSW)

		Gender		
		MSM *N*(%)	MSM *N*(%)	MSM *N*(%)
		259 (53)	233 (47)	492
Mean age in years (min-max)		29 (18–76)	35 (19–60)	32 (18–76)
Age classes				
18–25 CI 95		83 (33) 27,1%-39,1%	43 (19) 13,8%-24,2%	126 (26) 22,3%-30,1%
> 25–50 CI95		161 (64) 57,6%-69,8%	173 (75) 68,7%-80,3%	334 (69) 64,8%-73,1%
> 50–70 CI95		6 (2) 0,8%-5,1%	15 (6) 3,6%-10,4%	21 (4) 2,8%-6,5%
> 70 CI 95		2 (1) 0,1%-2,8%	0	2 (0.4) 0,1%-1,5%
Sexual Orientation	Homosexual CI 95	188 (73.0) 67,2%-78,4%	1 (0.4) 0,0%-2,3%	189 (38.6) 34,3%-42,9%
	Bisexual CI 95	68 (26.3) 21,1%-32,3%	9 (3.9) 1,7%-7,2%	77 (15.7) 12,7%-19,2%
	Heterosexual IC 95	–	211 (90.6) 86,0%-93,9%	211 (43.1) 38,7%-47,4%
	Undefined CI 95	1 (0.4) 0,0%-2,1%	12 (5.1) 2,6%-8,8%	13 (2.6) 1,5%-4,4%
Educational level	Illiterate CI 95	16 (6.1) 3,6%-9,9%	67 (28.8) 23,0%-35,0%	83 (16.9) 13,8%-20,5%
	Primary school CI 95	33 (12,8) 9,0%-17,5%	56 (24) 18,7%-30,0%	89 (18.2) 15,0%-21,8%
	Secondary CI 95	101 (39.3) 33,2%-45,5%	80 (34.3) 28,2%-40,8%	181 (37) 32,7%-41,3%
	University CI 95	107 (41.4) 35,5%-47,9%	30 (12.9) 8,8%-17,8%	137 (27.9) 24,1%-32,0%
Employment Status	Unemployed CI 95	79 (30.9) 25,1%-36,7%	123 (52.8) 46,1%-59,3%	202 (41.2) 36,9%-45,6%
	Nonformal employmentCI 95	35 (13.9) 9,6%-18,4%	62 (26.6) 21,0%-32,7%	97 (20) 16,5%-23,5%
	Civil servant CI 95	51 (19.7) 15,1%-25,2%	15 (6.4) 3,6%-10,4%	66 (13.4) 10,7%-16,7%
	Liberal profession CI 95	35 (13.5) 9,6%-18,4%	20 (8.6) 5,3%-12,9%	55 (11.2) 8,7%-14,3%
	Student CI 95	57 (22) 17,2%-27,7%	13 (5.6) 3,0%-9,3%	70 (14.2) 11,4%-17,6%
Relationship Status	Single	239 (93) 89,1%-95,8%	99 (42.4) 36,0%-49,1%	338 (68.9) 64,7%-72,9%
	Married	10 (3,8) 1,8%-7,0%	6 (2.5) 0,9%-5,5%	16 (3.2) 2,0%-5,2%
	In relationship with a stable partner	7 (2,72) 1,1%-5,5%	6	13 (2,65) 1,5%-4,4%
	Divorced/separated	1 (0,3) 0,0%-2,1%	107 (45.9) 0,9%-5,5%	108 (22,0) 18,6%-25,9%
	widowed	0 (0)	15 (6.4) 3,6%-10,4%	15 (3.0) 1,8%-4,9%

### Acceptability and usability of oral HIVST

We record a very high rate of acceptability of the HIVST, 90,6% of our population, both MSM and FSWs, would be ready to use it and buy it if the price was around 50 MAD (IQR = 50) which is equivalent to 5€.

We found that only 18,1% of participants were aware of the existence of the self-test before participating in this study (see Table 2).

**Table 2:** Acceptability of HIVST key populations including MSM and FSW living in Morocco (*n* = 492)

		MSM *N*(%)	FSW *N*(%)	Total *N*(%)
Acceptability (Willing to buy and to use HIVST if available)	Yes CI 95	233 (90,6) 86,4%-93,9%	211 (90,5) 86,0%-93,9%	444 (90,6) 87,7%-92,8%
	NO CI 95	24 (9,3) 6,0%-13,5%	21 (9,0) 5,6%-13,4%	45 (9,1) 6,9-12,0%
Knowledge of HIV self-testing	Yes CI 95	76 (29.3) 24,0%-35,5%	13 (5.6) 3,0%-9,3%	89 (18,1) 15,0%-21,8%
	No CI 95	183 (70.7) 64,4%-75,9%	220 (94.4) 90,6%-97,0%	403 (81,8) 78,1%-85,0%
Performing the HIV self-diagnosis test	Very easy	237 (92.2) 88,2%-95,1%	188 (80.6) 75,0%-85,5%	425 (86.7) 83,4%-89,4%
	Rather easy	15 (5.8) 3,3%-9,4%	34 (14.5) 10,3%-19,7%	49 (10) 7,6%-12,9%
	Rather difficult	4 (1.6) 0,4%-3,9%	6 (2.5) 0,9%-5,5%	10 (2,0) 1,1%-3,7%
	very difficult	1 (0.3) 0,0%-2,1%	5 (2.1) 0,7%-4,9%	6 (1.2) 0,5%-2,6%
Interpretation of the test results	Very easy	231 (89,8) 85,5%-93,2%	186 (79.8) 74,0%-84,7%	417 (85.1) 81,6%-87,9%
	Rather easy	19 (7.3) 4,5%-11,3%	37 (15.8) 11,4%-21,2%	56 (11.4) 8,9%-14,5%
	Rather difficult	6 (2.3) 0,8%-5,0%	6 (2.5) 0,9%-5,5%	12 (2.4) 1,4%-4,2%
	very difficult	1 (0.3) 0,0%-2,1%	4 (1.7) 0,4%-4,3%	5 (1,2) 0,4%-2,3%

Performing the salivary HIV self-test was considered very easy for 92,2% of MSM versus 80,6% of FSW. Of the 6 people who found it very difficult to perform the self-test, 5 were FSW, 4 of them could not read or write.

### Bivariate analysis for level of ease in performing performing HIVST

In the univariable analysis, age, being a female sex worker, heterosexual orientation, being illiterate or having primary school level, not knowing about HIVST before and having difficulty to interpret HIVST were associated with having difficulty to perform HIVST. Being student or having a liberal profession were negatively associated with having this difficulty (Table 3).

**Table 3:** Bivariate analysis for performing HIVST

Variable	Odd ratio	Lower 95% C.I.for EXP(B)	Upper 95% C.I.for EXP(B)	*P* value
AGE	1,0	1,0	1,0	0,010
Key population				
MSM	1,0			
FSW	2,8	1,6	4,9	0,000
Sexual orientation				
Homosexual	1,0			
Bisexual	1,2	0,4	3,2	0,645
Heterosexual	3,1	1,6	5,8	0,001
Undefined	2,2	0,4	11,2	0,315
Education				
Illetriate or primary school	4,2	1,4	12,52	0,008
Secondaryor University	1,0			
Profession				
Unemployed	1,0			
Nonformal employment	1,3	0,7	2,4	0,371
Civil servant	0,0	0,0		0,997
Liberal profession	0,4	0,1	1,2	0,142
Student	0,2	0,0	0,8	0,023
Knowledge of HIVST				
Yes	1,0			
No	1,6	0,7	3,6	0,193

### Multivariate analysis for level of ease in performing performing HIVST

**Fig. 3:**
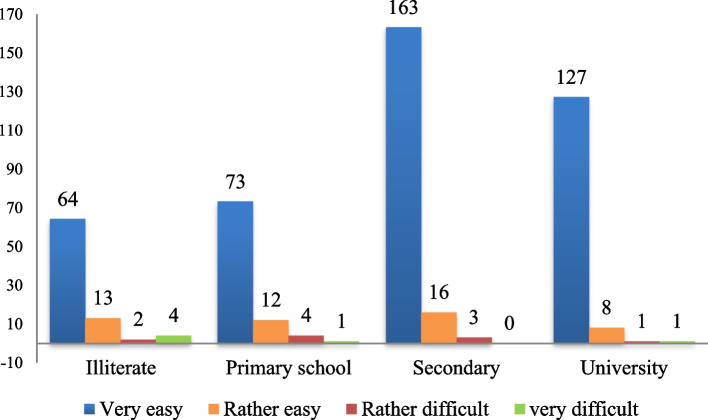
Usability of HIV Self-Testing by Education level

In the multivariate analysis only a low education level was significantly associated with having difficulty to perform HIVST (Table 4). What can explain that the interpretation of the OraQuick HIV Saliva Self-Test was more difficult among FSW with primary education or illiteracy. (Fig. 3).

**Table 4:** Multivariate analysis for performing HIVST

Variable	Odd ratio	Lower 95% C.I.for EXP(B)	Upper 95% C.I.for EXP(B)	*P* value
Key population				
MSM	1,0			
FSW	1,5	0,5	4,9	0,436
Education				
Illetriate or primary school	3,6	1,1	11,4	0,027
Secondaryor University	1000

### Positive rate and concordance between HIVST and conventional blood test results

Based on participants’ interpretation of the HIVST, 18 participants were tested positive among the 492 participants, 6 FSW and 12 MSM. The results of the standard rapid blood test performed by ALCS lay provider HIV testing confirmed these results resulting in 100% concordance between the 2 tests and a global HIV-positive rate of 3.7, 4.6% in MSM, and 2.6% in FSW.

## Discussion

In this pilot study, we aimed to assess the acceptability and feasibility of HIVST for FSWs and MSM and we evaluated the usability of a salivary HIVST, the OraQuick Rapid HIV-1/2 Antibody HIV self-test, in Morocco, according to WHO recommendations for the usability assessment of self-testing tools [25].

We report a high level of HIVST acceptability (90,6%) within the key populations, all the participants showed interest in using HIVST if it was available, and they would be ready to buy it if the price was around 50 MAD on average, which is equivalent to 5€.

Our findings are on acceptability are similar to recent results of pilot studies conducted in Kenya and the Democratic Republic of the Congo, with acceptability rates of 94 and 98% respectively [18, 26], showing high acceptability and uptake of HIVST in key populations.

Only 18,1% of participants were aware of the existence of the HIVST before participating in this study, 5,6% in FSW Vs 29,3% in MSM. Participants’ low level of knowledge was expected since HIVST is a relatively new screening method that is not yet available in the country. That low awareness of HIVST is also consistent with results from other studies conducted in low and middle-income countries [13].

Most participants (86.7%) deemed performing HIVST was very easy, and the vast majority (96.5%) have found that the interpretation of HIVST was rather easy or very easy. Our findings are similar to other studies such as in Benin, in sub-Saharan Africa [32], in the Democratic Republic of the Congo. [33].

However, performing OraQuick HIV Saliva Self-Test was more difficult among FSW. Indeed among the 6 people who found it very difficult to perform, 5 were FSW and it is noteworthy to stress that 4 out of the 5 could not read or write.

Our findings are consistent with many studies conducted in several contexts, which have shown that difficulties in performing HIV testing are particularly related to low levels of education [13, 26–28], particularly among FSW [29, 30].

Furthermore, we report that 18 participants were tested HIV positive, 12 are MSM (4.6%), and 6 are FSWs (2.6%). This high seropositivity among MSM is in agreement with national HIV prevalence among MSM is 4.9 [23].

Following the positive results of this study confirming the usability of this test, two other pilot studies were launched by the ALCS in collaboration with the Ministry of Health to assess the relevance of two different distribution approaches:1- peer-led distribution and, 2-Online request, and recovery in partner pharmacies. (Results not yet published).

In 2021 OraQuick HIV-1/2 rapid antibody test obtained authorization for sale and currently HIVST is available in some pharmacies in addition to remaining still available for ALCS beneficiaries belonging to key populations for free.

## Limitations

This study is subject to 2 limitations. First, the re-reading of the HIVST result by the lay provider HIV testing could lead to errors in the interpretation of the tests, because it has been reported that delayed re-reading of used OraQuick HIV-1/2 rapid antibody tests is not currently a valid methodological approach to quality assurance and may overestimate true HIV-positivity [31]. The reason why all the participants had another blood test performed simultaneously by the lay provider HIV testing is to ensure the validity of the results. We also made sure that the rereading did not exceed 20 min.

The second limitation that we acknowledge is that we have used only the oral HIV self-tests, and hence, unfortunately, our results do not encompass all forms of HIV self-testing, such as blood-based HIVST.

## Conclusion

In conclusion, our study shows very high acceptability for HIVST among FSWs and MSM in Morocco. HIV self-testing is still largely unknown by key populations in Morocco, and the low level of education of FSWs may be a barrier to the use of this test, but with the proposed assistance and adapted demonstration tools, the HIV self-testing will certainly improve access to testing in Morocco. Moreover, HIVST offer should be integrated with prevention services and programs that promote safer sex practices.

## Data Availability

The datasets generated and/or analyzed during the current study are not publicly available due to the inclusion of potentially identifying and sensitive information but are available from the corresponding author on reasonable request.

## Abbreviations

ALCS: Association de Lutte Contre le Sida
FSW: Female Sex Workers
HIV: Human Immunodeficiency Virus
HIVST: HIV self-testing
MAD: Dirham Marocain
MENA: the Middle East and North African
MSM: Men who have Sex with Men
PLHIV: People living with HIV
PrEP: Pre-Exposure Prophylaxis
UNAIDS: The Joint United Nations Programme on HIV and AIDS UNITAID: Global Health Agency
WHO: The World Health Organisation
