# Correction: Acceptability and usability of oral fluid-based HIV self-testing among female sex workers and men who have sex with men in Morocco

**DOI:** 10.1186/s12889-022-14955-3

**Published:** 2022-12-28

**Authors:** Amal Ben Moussa, Ouijdane Belhiba, Fatima Zahra Hajouji, Amina El Kettani, Mohammed Youbi, Kamal Alami, Boutaina El Omari, Lahoucine Ouarsas, Mehdi Karkouri

**Affiliations:** 1 Association de Lutte Contre le Sida (ALCS), Casablanca, Morocco; 2 Community-based Research Laboratory, Coalition PLUS, Pantin, France; 3 Direction de l’Epidémiologie et de Lutte contre les Maladies (DELM), Rabat, Morocco; 4 Joint United Nations Programme on HIV and AIDS (UNAIDS), Rabat, Morocco

**Correction: BMC Public Health 22, 2266 (2022)**

**https://doi.org/10.1186/s12889-022-14632-5**

The original publication of this article [1] contained 2 errors which were introduced during the publication process:

1. The headings of table 1 were incorrect as it listed “MSM” three times.
2. The last 2 lines of table 3 were missing “IC95”

The original article has been updated to correct these errors.
